# Tailoring the Design of Dendritic Thermogels Through Carbosilane and Polyglycerol Crosslinkers

**DOI:** 10.3390/pharmaceutics18030362

**Published:** 2026-03-13

**Authors:** Judith Recio-Ruiz, Boonya Thongrom, F. Javier de la Mata, Rainer Haag, Sandra García-Gallego

**Affiliations:** 1 University of Alcala, Department of Organic and Inorganic Chemistry and Research Institute in Chemistry “Andrés M. Del Río” (IQAR), 28805 Madrid, Spain; judith.recio@uah.es (J.R.-R.); javier.delamata@uah.es (F.J.d.l.M.); 2 Institute for Chemistry and Biochemistry, Freie Universität Berlin, Takustr. 3, 14195 Berlin, Germany; boonya.th@hotmail.com (B.T.); haag@zedat.fu-berlin.de (R.H.); 3 Networking Research Center on Bioengineering, Biomaterials and Nanomedicine (CIBER-BBN), 28029 Madrid, Spain; 4 Institute Ramón y Cajal for Health Research (IRYCIS), 28034 Madrid, Spain

**Keywords:** dendrimer, carbosilane, polyglycerol, hydrogel, thermogel, temperature, drug release, doxorubicin

## Abstract

**Background/Objectives:** The development of stimuli-responsive hydrogels for biomedical uses is an intense field of research. The use of dendritic crosslinkers can enhance the control over the structure and properties of the networks. This work presents a comparative study on the design and evaluation of Pluronic L35 thermogels, incorporating either hydrophobic carbosilane dendrimers (CBS, generations 1 to 3) or hydrophilic dendritic polyglycerols (dPG, 10 k) as crosslinkers. **Methods:** The thermogels were synthesized via UV-initiated thiol–ene click chemistry. Additionally, they were characterized through swelling studies, mechanical properties, degradation kinetics as well as loading and release studies of the antitumor drug doxorubicin as poorly soluble model cargo. **Results:** The incorporation of dendritic crosslinkers allowed higher control over the crosslinking process, while the amphiphilic polymer imparted temperature-responsive properties to the resulting networks. Remarkable differences were observed in swelling behavior, mechanical properties and degradation kinetics, depending on the nature of the dendritic crosslinker. Additionally, regarding doxorubicin loading and release in water, CBS hydrogels produced a sustained release over one week, led by network swelling, while dPG hydrogels exhibited a burst release in 4–24 h but were limited by the stronger interaction of DOX with the dPG scaffold. **Conclusions:** The study provided useful insight for the tailoring of dendritic thermogels for specific biomedical uses such as controlled drug delivery.

## 1. Introduction

Nowadays, there is intense research towards the development of stimuli-responsive hydrogels for biomedical uses. These “smart” hydrogels exhibit changes in their structure, properties, or behavior in response to specific stimuli like pH or temperature [1]. In particular, thermo-responsive hydrogels or “thermogels” merge the high water content and tunable properties of hydrogels with the ability to respond to external temperature change with a simple, physical and reversible sol-to-gel phase transition [2]. Typically, they are formed by a polymer in a solution state, which gels at higher temperatures due to hydrophobic interactions [3]. It has been described that the final gelling mechanism is due to micelle aggregation. The minimum concentration and temperature required for the micelle-to-hydrogel transition are denoted as “critical gelation concentration” (CGC) and “critical gelation temperature” (CGT), respectively. Three different packing mechanisms have been described [2]: (1) individual micellar packing, like the one occurring in short-chain Pluronic polymers, where the entanglement of the hydrophilic part forms a physical crosslinking; (2) inter-micellar bridged packing, happening, for example, in BAB copolymers, where the hydrophobic segments are connected to two or more micelles; and (3) micellar corona collapse packing, happening in polymers with low LCST, like PNIPAM.

Thermogels are often classified as biodegradable, like the ones formed by polyesters, polypeptides or polysaccharides, or non-biodegradable, like those based on polyacrylates or Pluronic^®^ polymers. Pluronics are a family of amphiphilic block copolymers, broadly used in pharmaceutical and clinical applications due to their low immunogenicity and exceptional biocompatibility [4]. Their uses as surfactants, solubilizers, emulsifying agents and absorption enhancers have been reported, as well as their temperature-dependent gelation and micellization [5]. Despite the promising properties of Pluronic gels, for example, for injectable formulations, it has been reported that they present poor mechanical properties and are easily eroded after administration due to immediate dilution [6]. To increase the stability of Pluronic hydrogels, different approaches that combine the thermo-responsive sol–gel transition of Pluronics with other materials with crosslinking capabilities have been explored. For example, Pouso et al. designed a dual-crosslinked Pluronic F127–chitosan hydrogel, which benefited from the ionotropic gelation of chitosan in the presence of NaHCO_3_ and prevented the premature degradation of Pluronic hydrogels [7]. Ulanski et al. explored the radiation-induced crosslinking of Pluronic F127 [8]. They observed the intermolecular recombination of Pluronic radicals as well as chain scission, as well as a clear dependence of the radiolysis mechanism on the spatial arrangement of Pluronic molecules.

A more robust and controlled approach to stabilize Pluronic hydrogels relies on the use of dendritic crosslinkers to generate dendritic networks [9]. Dendritic hydrogels are a growing field that is poised for impactful translation advances in biomedicine. This field has evolved from proof-of-concept studies to the design of advanced application-driven solutions. The initial works in the early 2000s explored the ability of dendritic macromolecules to form supramolecular networks. For example, Caminade and Majoral showed the outstanding gelation ability of organophosphorus dendrimers at very low concentration [10]. Likewise, Grinstaff designed dendritic–linear hybrids with photo-crosslinkable properties and employed them to seal corneal lacerations [11]. These seminal works are representative examples of the two main synthetic approaches towards dendritic hydrogels: the formation of physical networks or the use of covalent crosslinking through diverse chemistries like thiol–ene “click” chemistry, photo-polymerization, aza-Michael addition, and strain-promoted azide–alkyne cycloaddition (SPAAC), among others.

The role and nature of the multifunctional dendritic crosslinker are crucial in this process. Carbosilane dendrimers, with a lipophilic nature, have recently been employed to generate amphiphilic hydrogels for different purposes. For example, Muñoz-Sánchez et al. employed UV-initiated thiol-ene coupling to generate different dendritic hydrogels based on Pluronic L31, L35 and L61, among other polymers [9]. The study confirmed the impact of the linear polymer on the amphiphilic properties of the dendritic network, affecting the swelling, thermo-response and drug delivery. Furthermore, the use of carbosilane dendrons as crosslinkers provided functional networks that could be precisely modified with fluorescent dyes. Altering the carbosilane crosslinkers enables a precise control on the hydrogel properties, providing, for example, improved interaction with aromatic drugs [12]; modulable antibacterial properties [13]; or controllable network degradation [14].

Dendritic polyglycerol (dPG) has also been broadly studied as a crosslinker to generate hydrogels for biomedical applications, such as protein delivery from nano- and microgels and cell-encapsulation and release from microgels and bulk hydrogels. For example, Thongrom et al. designed dPG-PEG hydrogels via thiol-click reactions for biosensing purposes [15]. dPG was functionalized with either an acrylate, allyl or acrylamide group and reacted with a dithiolated PEG 6 k, to obtain a library of tailor-made hydrogels to modulate streptavidin encapsulation. All the hydrogels exhibited promising activity as biosensing platforms, but with important differences regarding their degradability. On a smaller scale, Sisson et al. designed azide-functional dPG to form microgels via CuAAC for cell encapsulation [16] and Steinhilber et al. prepared pH-cleavable cell-laden microgels using SPAAC and droplet-based microfluidics, employing azide–dPG and PEG–dicyclooctyne as precursors [17]. Dendritic polyglycerol has also been employed for the design of thermo-responsive nanogels, using poly(N-isopropylacrylamide) as a complementary polymer [18]. The authors demonstrated that, at temperatures higher than 35 °C, the particle size instantly collapsed, reducing the size and releasing the loaded cargo. This supports the interest in the development of thermo-responsive gels based on dPG.

Despite advances in dendritic hydrogels, comparative studies evaluating the influence of different dendritic crosslinkers, with different hydrophilic–hydrophobic properties, on thermoresponsive properties and drug release behavior remain limited. In this work, we designed and evaluated Pluronic L35-based dendritic thermogels incorporating hydrophobic carbosilane dendrimers (CBS) or hydrophilic hyperbranched polyglycerol (dPG) as crosslinking agents. The networks were synthesized via UV-initiated thiol–ene chemistry and characterized through swelling studies, mechanical properties, degradation, as well as loading and release of doxorubicin as a model of a poorly soluble drug.

## 2. Materials and Methods

### 2.1. Materials

All reagents and solvents were purchased from commercial sources with purity ≥ 95% and used as received without further purification. Pluronic L35, 3-mercaptopropionic acid and *p*-toluenesulfonic acid monohydrate were purchased from Sigma Aldrich (St. Louis, MO, USA). 2,2-dimethoxy-2-phenylacetophenone (DMPA) was purchased from Acros Organics (Geel, Belgium). Chloroform was purchased from J.T. Baker (Phillipsburg, NJ, USA). Acetone, tetrahydrofuran, and methanol were purchased from Sigma Aldrich in HPLC grade. Previously reported synthetic procedures were used for PLUL35(SH)_2_ [9], the vinyl-decorated carbosilane dendrimers **1***–***3** [19] and the acrylate- and allyl-functional hyperbranched polyglycerols **4–5** [15].

### 2.2. General Methods

NMR spectroscopy. NMR spectra were acquired at CAIQ-UAH, using the Varian NMR System-500 (Varian, Inc., Palo Alto, CA, USA), Varian Mercury Plus-300 and Bruker AVANCE Neo 400 instruments (Bruker, Billerica, MA, USA) at room temperature and using CDCl_3_ as the solvent. The chemical shifts are expressed in ppm using the solvent as an internal reference in ^1^H-NMR (CDCl_3_ δ (^1^H) = 7.24 ppm).

High-Performance Liquid Chromatography (HPLC). DOX release studies were performed at CAIQ-UAH on Agilent 1200 HPLC equipment (Agilent, Santa Clara, CA, USA), using an ACE Excel 5 C18 250 mm × 4.6 mm column (Advanced Chromatography Technologies Ltd., Aberdeen, UK) and a mobile phase of 0.1% trifluoroacetic acid and acetonitrile (40:60), with an injection volume of 10 μL. DOX was detected at a wavelength of 234 nm.

Rheology. The viscoelastic properties of hydrogels were measured using the following: (1) Carbosilane hydrogels: A Discovery Hybrid Rheometer 10 (DHR-10) from TA Instruments (New Castle, DE, USA); and (2) Polyglycerol hydrogels: A Kinexus rheometer from Malvern Instruments (Malvern, UK). Both instruments were equipped with a parallel-plate geometry (8 mm diameter). Amplitude sweep (0.1–100% strain at 1 Hz) and frequency sweep (0.1–10 Hz at 1% strain) tests were performed at 25 °C and 37 °C. All measurements were performed in duplicate in two independent experiments.

Dynamic Light Scattering. The hydrodynamic diameters of the micellar systems were obtained with a Zetasizer Nano ZS (Malvern Instruments Ltd., Malvern, UK) at CAIQ-UAH. Measurements were performed in disposable plastic cuvettes at 25 °C and 37 °C.

Hydrogel degradation study. The hydrogel was immersed in phosphate-buffered saline (PBS, Sigma-Aldrich, St. Louis, MO, USA; 600 μL, pH 7.4) and incubated for 15 days at 37 ± 0.5 °C. The change in storage modulus (G′) was measured over time through rheological tests at 37 °C.

### 2.3. Synthesis of Carbosilane Dendritic Hydrogels

The dendrimer (**1**, **2** or **3**) was dissolved in the minimum amount of THF/MeOH (1:2) at 150 mg/mL, and the mixture was degassed with argon. PLUL35(SH)_2_ (SH/alkene 1:1) and DMPA (5% mol/alkene) were added. The reaction mixture was stirred gently until complete dissolution of the reagents and then exposed to UV light (365 nm, 30 W) for 90 min. After crosslinking, the gel was removed from the lamp, dried under vacuum, and weighed. The hydrogels were purified by washing with acetone under orbital shaking until complete removal of DMPA was observed by TLC.

### 2.4. Synthesis of Polyglycerol Dendritic Hydrogels

The hyperbranched polymer **4** or **5** was dissolved in the minimum amount of THF/H_2_O (1:2) at a concentration of 150 mg/mL, and the mixture was degassed with argon. PLUL35(SH)_2_ (SH/alkene 1:1) and LAP (lithium phenyl-2,4,6-trimethylbenzoylphosphinate, Sigma-Aldrich, St. Louis, MO, USA, 5 mg/mL) were added. The reaction mixture was stirred gently until complete dissolution of the reagents and then exposed to UV light (365 nm, 36 W) for 2 h. After crosslinking, the gel was removed from the lamp. The hydrogels were used without further purification.

### 2.5. Characterization of Dendritic Hydrogels

Gel Fraction (GF%). After UV crosslinking, the obtained hydrogels were dried under vacuum until constant weight was achieved (W_UV_). The samples were then washed with acetone under orbital shaking to remove unreacted species and the catalyst. After purification, the hydrogels were dried again under vacuum (W_D_). The gel fraction (GF%) was calculated using Equation (1) [20]:

(1) $$\mathrm{GF}\%\;=\;\frac{W_{D}}{W_{UV}}\times 100$$

where W_UV_ is the weight of dry mass after the UV reaction, and W_D_ is the weight of dry mass after purification. All measurements were performed in duplicate in two independent experiments.

Swelling Degree (SD%). Carbosilane hydrogels were immersed in distilled water for 10 days. Periodically, they were removed, and their size and weight were measured. Swelling experiments were performed at 25 ± 0.5 °C. All gels were tested in duplicate in two independent experiments. The SD is calculated with the following equation [20]:

(2) $$\mathrm{SD}\%\;=\;\frac{\left(W-W_{D}\right)}{W}\times 100$$

where W is the weight of the swollen gel, and W_D_ is the dry gel mass after purification.

Mesh Size. The mesh size of the hydrogels was calculated from the storage modulus measured in frequency sweep assay at 1 Hz, using the following equation from the classical theory of rubber elasticity [21,22]:

(3) $$r\;=\;({\frac{6RT}{\pi N_{AV}G})}^{1/3}$$

where r is the mesh size (nm), R is the gas constant (8.314 m^3^ Pa K^−1^ mol^−1^), T is the temperature (K), N_AV_ is Avogadro’s number (6.022 × 10^23^ mol^−1^), and G is the storage shear modulus (Pa).

### 2.6. Doxorubicin Loading and Release Assays

DOX loading in carbosilane hydrogels. The selected hydrogel was immersed in 1 mL of a saturated solution of doxorubicin (Sigma-Aldrich, St. Louis, MO, USA, 5 mg) in ethanol and exposed for 30 min at r.t. under light orbital shaking. Then, the gel was removed from the vial and dried under vacuum. The remaining DOX in the solution was quantified through HPLC to calculate the encapsulated DOX. Hydrogels’ average weight and loaded DOX: **H2** (32.0/0.13 ± 0.02 mg) and **H3** (47.6/0.23 ± 0.02 mg).

DOX loading in polyglycerol hydrogels. The dendritic crosslinker was dissolved in the minimum amount (500 µL) of THF/H_2_O (1:2), and the mixture was degassed with argon. PLUL35(SH)_2_ (SH/ene 1:1), LAP (5 mg/mL) and DOX (5 mg/mL) were added. The reaction mixture was stirred gently until complete dissolution of the reagents and then exposed to UV light (365 nm, 36 W). After crosslinking, the gel was removed from the lamp. The remaining DOX in the solution was quantified through HPLC to calculate the encapsulated DOX. Hydrogels’ average weight and loaded DOX: **H4** (50.8/0.54 ± 0.03 mg) and **H5** (68.1/0.22 ± 0.02 mg).

DOX release assays. DOX-loaded hydrogels were immersed in 1 mL of distilled water (pH 7.4) and incubated at 37 ± 0.5 °C under orbital agitation. At predetermined time intervals, an aliquot of the release medium was collected for analysis and replaced with an equal volume of distilled water. The amount of released DOX was quantified by HPLC using the conditions described above. Sink conditions were maintained during the release experiments, as the maximum theoretical concentration of doxorubicin in the release medium (~0.0975 mg/mL) remained below 10% of its aqueous solubility at 37 °C.

## 3. Results

### 3.1. Synthesis and Characterization of Dendritic Thermogels

**Figure 1:**
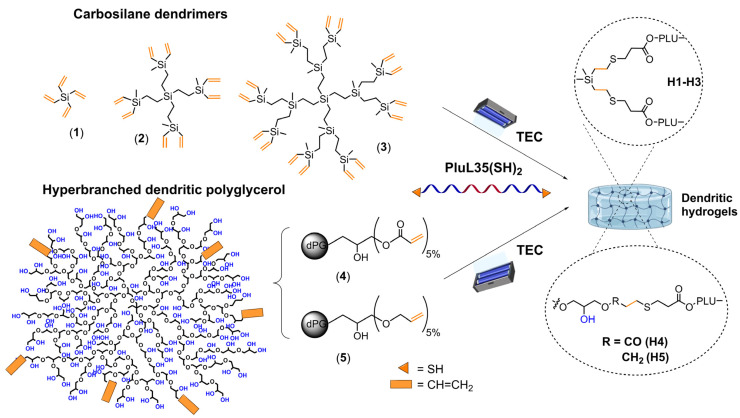
Synthesis of dendritic thermogels **H1**–**H5** through UV-initiated thiol–ene chemistry. Vinyl-functional carbosilane dendrimers (**1**–**3**) and polyglycerol hyperbranched polymers bearing 5% of acrylate (**4**) or allyl (**5**) groups were used as crosslinkers, together with the amphiphilic polymer PLUL35(SH)_2_. For CBS hydrogels, DMPA was used as a photoinitiator, with 1.5 h of irradiation. For dPG hydrogels, water-soluble LAP was used as a photoinitiator, with 2 h of irradiation.

Photo-activated thiol-ene click (TEC) chemistry is a broadly employed tool for the design of polymeric networks. It is robust, versatile, and presents simple reaction conditions. TEC was employed herein as the main tool for the synthesis of dendritic hydrogels. Accordingly, we used an alkene-functional dendritic crosslinker and the amphiphilic polymer Pluronic L35, modified in the terminal units with thiol groups PluL35(SH)_2_ (Figure 1). This polymer was synthesized as recently reported [9]. Traditional vinyl-functional carbosilane dendrimers SiGnV_m_ (**1**–**3**) and polyglycerol hyperbranched polymers with pendant acrylate and allyl groups (**4**, **5**) were also synthesized as previously reported [15,19]. In brief, using hPG_10k_-OH as precursor, 5% of acrylate groups were introduced through reaction with acryloyl chloride, to obtain hPG_10k_(OH)(ac) (**4**). Similarly, 5% of allyl groups were introduced with allyl bromide, to obtain hPG10k(OH)(al) (**5**). Both were characterized by ^1^H-NMR end group analysis (Figures S1 and S2).

Despite our desire to obtain a general protocol for the synthesis of the dendritic networks, the distinct physicochemical properties of the CBS and dPG crosslinkers, including solubility, hydrophilic–hydrophobic balance, and reactivity of functional groups, led to a specific optimization of the synthetic approach for each family of thermogels. These two approaches required the use of different solvents, photoinitiators, and purification procedures to achieve effective network formation, somewhat limiting an accurate comparison of CBS and dPG thermogels. Hence, the following discussion must be analyzed in qualitative terms between the two families, while quantitatively within each one.

#### 3.1.1. Carbosilane Dendritic Thermogels

CBS thermogels were synthesized through a previously reported protocol [9]. In brief, the selected dendrimer and PluL35(SH)_2_ (thiol:alkene ratio 1:1) were dissolved in a THF:MeOH mixture (1:2) and exposed to UV light for 90 min in the presence of the photoinitiator DMPA (5% mol/alkene). Then, the hydrogels were thoroughly washed with acetone to remove DMPA and unreacted species. To evaluate the impact of the dendrimer generation, three different hydrogels were prepared: **H1**, **H2** and **H3**, using dendrimers SiG0V_4_ (**1**), SiG1V_8_ (**2**) and SiG2V_16_ (**3**), respectively. Main parameters are summarized in Table 1.

**Figure 2:**
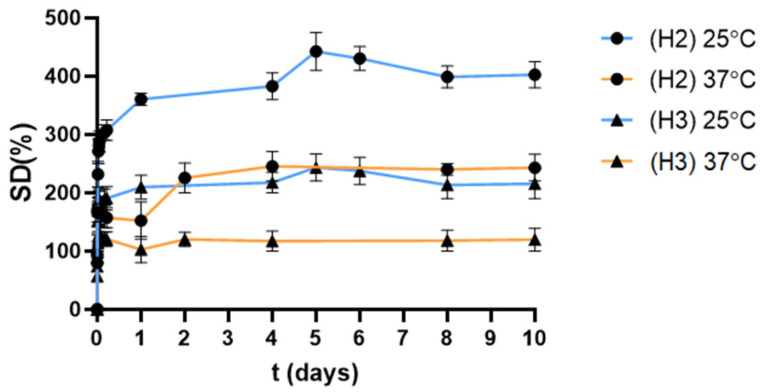
Change in swelling degree over time for carbosilane hydrogels **H2** and **H3**, at 25 and 37 °C. Results are reported as mean ± standard deviation, in duplicate for two independent experiments.

All hydrogels were characterized through their gel fraction (GF%) and the swelling degree (SD%), Table 1. The GF%, which is calculated through Equation (1), provides an indirect measurement of the efficacy of the crosslinking reaction. Higher gel fraction values indicate a greater extent of covalent network formation and a lower soluble fraction, which is consistent with higher reaction conversion. After 90 min of UV irradiation, **H1** obtained 95% GF, thus confirming the efficacy of thiol–ene crosslinking reaction with the smaller crosslinker SiG0V_4_ (**1**). Under the same conditions, higher generation crosslinkers **2** and **3** generated hydrogels **H2** and **H3** with GF ~60%. This showed that the increase in generation—and subsequently size, number of functional groups and lipophilic nature— requires longer reaction times to get all peripheral groups reacted. This agrees with our previous studies, which showed that **H2** required 4 h of irradiation to accomplish GF 90% [9]. Nevertheless, **H2** and **H3** showed enough stability to perform subsequent studies and were prepared under these conditions for comparative purposes. SD% is a relevant parameter that informs about the ability of the hydrogel to allocate solvent molecules in its interior, and it is calculated through Equation (2). Hydrogels **H1**–**H3** were immersed in water at both 25 °C and 37 °C, and their weight and size were measured over time. As depicted in Figure 2, hydrogels **H2** and **H3** underwent a similar pattern, with a fast swelling in the first 30–40 min and a steady plateau afterwards. Unfortunately, and despite its high GF, the nature of **H1** hindered the measurement of SD%, as it easily broke in multiple pieces during the assay.

Despite their similar gel fraction, **H2** and **H3** showed very different SD, which decreased with higher dendrimer generation. This could be ascribed to the scaffolds being turned more hydrophobic, and the adsorption of water molecules became less favorable. Furthermore, this study confirmed the temperature-responsive behavior of these hydrogels. As shown in Figure 2, hydrogels **H2** and **H3** shrank when the temperature was increased from 25 °C to 37 °C due to the nanostructuring of the hydrogels. As previously described, short-chain Pluronics like L35 can undergo individual micellar packing when increasing the temperature, as the hydrophilic part entangles and forms a physical crosslinking. In our case, the freedom of the hydrophilic part is restricted due to the thioether bonds, but the chains change their conformation, leading to around 50% shrinkage of the hydrogel at 37 °C. It is also worth noting that **H2** with GF 57% achieved higher swelling than that with GF 90%, previously described [9]. As expected, a lower crosslinking density of the network facilitates water absorption.

To gain further understanding of the properties of the linear polymer PluL35(SH)_2_, which surely affect the properties of the hydrogels, we studied its self-assembly behavior through Dynamic Light Scattering (DLS). It has been reported that the Critical Micellar Concentration (CMC) for the precursor polymer Pluronic L35 is 5.3 mM [5]. In our experiments, we observed that the precursor polymer PLU L35 did not self-assemble at 25 °C or 37 °C at the range of concentrations tested (<6 mM), Figure S3. On the contrary, PluL35(SH)_2_ rapidly formed highly monodisperse aggregates with Ø 200 nm, which could be micelles (PDI 0.097, at 6.0 mM); see Figure S3. These aggregates decreased their size and increased their polydispersity when diluted. This study confirmed the clear impact of attaching mercaptopropionic groups to the terminal units of PluL35 in its self-assembly properties. This behavior has been previously observed with other Pluronic polymers [23]. The aggregation phenomenon can be translated to the dendritic hydrogels herein described and explains properties such as the response to temperature, through Pluronic coil-to-globule transition upon heating due to the dehydration of PPO blocks.

#### 3.1.2. Polyglycerol Dendritic Thermogels

For the synthesis of dPG hydrogels, the hyperbranched polymers dPG_10k_(OH)(ac) (**4**) or dPG_10k_(OH)(al) (**5**) were reacted with PLUL35(SH)_2_ (thiol:alkene ratio 1:1), in a THF:water mixture (1:2) using LAP (5 mg/mL) as photoinitiator. Crosslinking was performed under UV light for 2 h (Table 1), leading to hydrogels **H4** and **H5**. The obtained materials exhibited a translucent and fragile appearance. Unfortunately, these hydrogels fragmented rapidly into several pieces when exposed to solvents, so it hindered the correct evaluation of their GF% and their SD%. The rationale behind this behavior can be found in a less efficient crosslinking reaction due to (Figure S4) (1) the presence of only 5% alkene groups available for reaction with PLUL35(SH)_2_; (2) the potential H-bond formation between the mercaptopropionic terminal units in the polymer and the multiple hydroxyl groups in dPG scaffold, blocking the thiol–ene reaction; or (3) side reactions, as under these conditions, acrylate-functionalized dPG (**4**) can also undergo self-crosslinking homopolymerization, thus decreasing the efficacy of thiol–acrylate reaction. Indeed, previous studies reported that acrylate-functionalized dPG showed curing of 97 wt% after UV irradiation for 20 s, exemplifying the fast side-reaction [24].

### 3.2. Impact of the Dendritic Crosslinker on the Mechanical Properties of the Thermogels

Oscillatory shear rheology (OSR) was used to evaluate the viscoelastic behavior of the hydrogels at 25 °C and 37 °C using parallel plates of Ø 8 mm. In this technique, a range of oscillatory shear strains is applied to the hydrogel, and its response in terms of deformation and flow is measured. As main parameters, we studied the storage modulus (G′) and the loss modulus (G″). G′ measures the hydrogel’s ability to store elastic energy when subjected to mechanical strain. In other words, it indicates the resistance to deformation when strain is applied, and its ability to recover its original shape once that strain is removed. Therefore, it provides information about the stiffness/flexibility of a material. On the other hand, the loss modulus (G″) represents the portion of energy dissipated during material deformation and is related to the viscous part of the material’s behavior.

#### 3.2.1. Mechanical Properties of Carbosilane Thermogels

**Figure 3:**
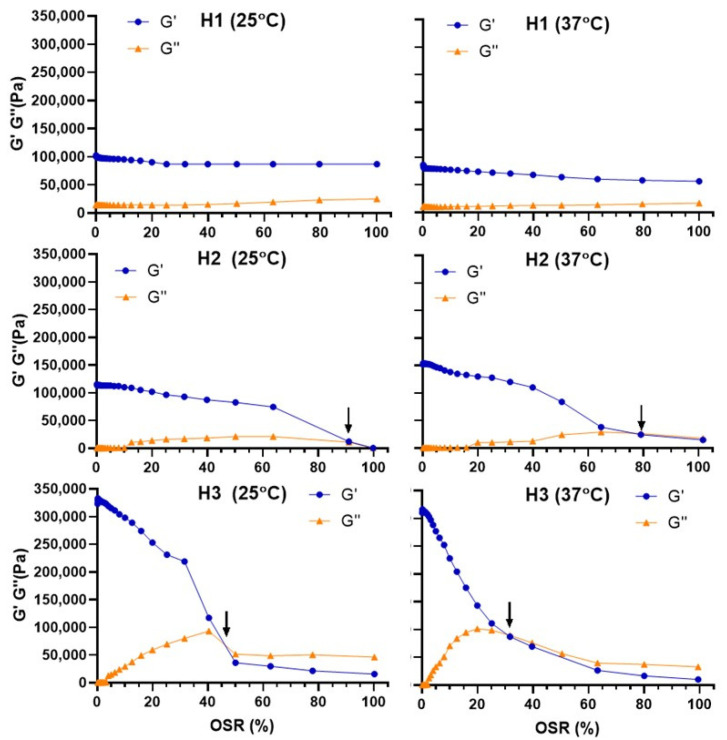
Amplitude sweep assay at 25 °C (**left**) and 37 °C (**right**) for carbosilane hydrogels **H1**–**H3**. The cross-over point is highlighted with an arrow. Results are reported as the mean from two independent experiments.

We initially defined the linear viscoelastic region (LVR) in amplitude sweeps where shear deformation is increased at a constant frequency. In this region, any applied deformation to the material will not result in any changes at the microstructural level. Strain sweeps also provided information about a crossover point when G′ = G″ and the material began to deform irreversibly. The results from the amplitude assay are depicted in Figure 3.

These assays revealed that carbosilane hydrogels are stable materials, requiring a very high strain to reach irreversible deformation. They showed an increased rigidity (higher G′) with increasing generation. This indicates that the irreversible deformation is reached at lower oscillation strains for higher generations. For example, at 25 °C, the crossover point is observed around 45% for **H3**, around 90% for **H2,** and it is not observed for the lower generation, **H1**. This also indicates that **H1** is less stiff but highly flexible and has the highest gel strength among them, while **H3** is the stiffest but brittle, seeing that the crossover point is the lowest among them. The increase in temperature also favors such irreversible deformation: at 37 °C, this point is observed around 30% for **H3**, around 80% for **H2**, and it is not observed for the lower generation **H1**. This is in line with the nanostructuring of Pluronic hydrogels, especially relevant at higher temperatures.

**Figure 4:**
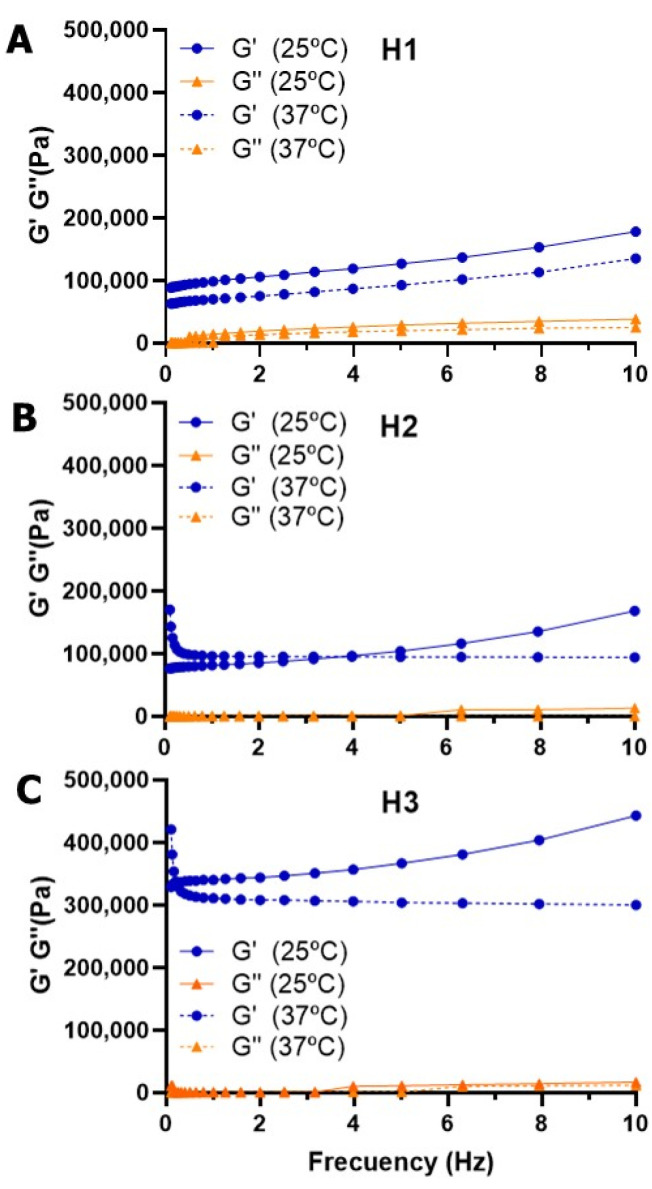
Frequency sweep for carbosilane hydrogels, at 25 °C and 37 °C. (**A**) Change in G’ and G’’ for hydrogel **H1**. (**B**) Change in G’ and G’’ for hydrogel **H2**. (**C**) Change in G’ and G’’ for hydrogel **H3**. Results are reported as the mean from two independent experiments.

Once the LVR was established, we performed a frequency sweep from 0.1 to 10 Hz at 1% constant strain (Figure 4). This assay confirmed the viscoelastic nature of our hydrogels, with G′ > G″ in all ranges, common for covalently crosslinked hydrogels. At 25 °C, an increase in G′ is observed with increasing frequency: at higher frequencies, the molecules have less time to reorganize and relax between deformation cycles, resulting in a stiffer and more elastic material response. Again, we confirmed the higher G′ for higher dendrimer generation, in agreement with the slight increase in GF% (Table 1) that results in more compact networks. The differences between **H1** and **H2** are practically negligible, but significant differences are found when transitioning to **H3**. Additionally, G′ and G″ values slightly change when the temperature is increased. Overall, we confirmed the remarkable impact of the dendrimer generation on the mechanical properties of the dendritic hydrogel, which allows the fine-tuning of the desired properties in the network.

#### 3.2.2. Mechanical Properties of Polyglycerol Thermogels

**Figure 5:**
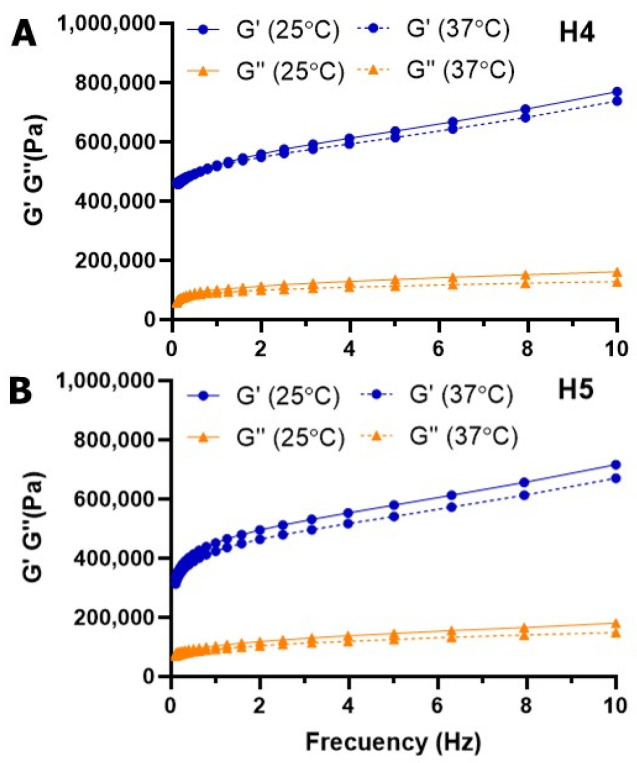
Frequency sweep for polyglycerol hydrogels, at 25 and 37 °C. (**A**) Change in G’ and G’’ for hydrogel **H4**. (**B**) Change in G’ and G’’ for hydrogel **H5**. Results are reported as mean from two independent experiments.

The results from the frequency sweep at 25 °C and 37 °C of polyglycerol hydrogels **H4** and **H5** are depicted in Figure 5. A similar behavior is found for both thermogels. The storage and loss moduli progressively increased with the frequency, confirming their viscoelastic behavior. Both exhibited higher G′ values than carbosilane hydrogels, which confirmed their stiffer nature. Among them, the acrylate-containing hydrogel **H4** is stiffer than the allyl-functional hydrogel **H5**, which could be ascribed to the self-crosslinking of acrylate groups on dendritic polyglycerol, which reinforces the hydrogel network. Furthermore, we found that the impact of temperature on their mechanical properties is less pronounced than in the carbosilane thermogels.

The mesh size of hydrogels **H1**–**H5** was estimated from the storage modulus value measured in the frequency sweep test at 1 Hz, using Equation (3), from the classical theory of rubber elasticity (Table 1) [21,22]. Carbosilane hydrogels presented a mesh size in the range 2.1–4.3 nm, while polyglycerol hydrogels were around 2.5 nm. These values are lower than those reported for similar dPG-PEG hydrogels, in the range 11–15 nm [15]. The different length of the polymer (PEG 6 k vs. PLU L35 2 k) as well as hydrophilicity (PEG is hydrophilic while Pluronic is amphiphilic) partially explains this difference, but a higher crosslinking density must also be considered, as well as stiffer behavior, a different molecular architecture, and the size of the crosslinker.

#### 3.2.3. Network Degradation Study

**Figure 6:**
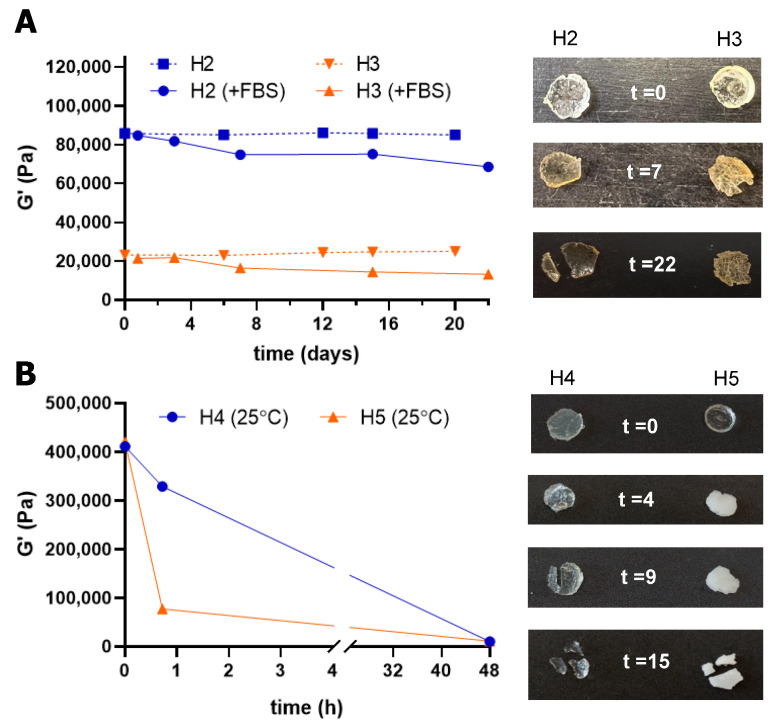
Degradation of thermogels over time, monitored through rheology. (**A**) CBS hydrogels **H2** and **H3** in either PBS or in the presence of esterases (in FBS at 37 °C). (**B**) dPG hydrogels at 25 °C in PBS. Results are reported as the mean from two independent experiments.

The degradation of the dendritic hydrogels in water solution was studied through rheology, monitoring the change in G′ over time. Carbosilane hydrogels **H2** and **H3** were stable in PBS for 20 days, as they showed no significant changes in G′ (Figure 6A). However, the presence of ester bonds in the network enabled a controlled cleavage in the presence of esterases of FBS, observing a progressive decrease in G′ over time. After 22 days, G′ decreased 20% and 40% for **H2** and **H3**, respectively, compared to the initial G′ value at t = 0. On the contrary, polyglycerol hydrogels **H4** and **H5** already exhibited a fast cleavage in PBS at 25 °C, showing a fast decrease in G′ even during the first minutes (Figure 6B). After only 40 min in PBS, the storage modulus for these hydrogels decreased around 20% for the acrylate-functional **H4** and 80% for the allyl-functional **H5**, compared to the initial G′ value at t = 0. This behavior can be potentially explained by their incomplete gelation due to side reactions. The analogous dPG-PEG hydrogels reported by Thongrom et al. exhibited quite different behavior [15]. The thiol–allyl hydrogel is stable for 11 days, at both 25 and 37 °C, and the thiol-acrylate hydrogel showed degradation from day 2 at 25 °C and a fast degradation at 37 °C. This highlights the impact of the linear polymer in the hydrogel network to control the stability and degradation pattern.

### 3.3. Doxorubicin Loading and Release Assays

To evaluate the potential of these hydrogels to control drug release, we selected doxorubicin (DOX) as a model antitumor drug. DOX is a well-known drug in clinical therapy against cancer, with multiple mechanisms including DNA damage, reactive oxygen species production, apoptosis, senescence and autophagy, among others [25]. However, DOX also exhibits adverse effects like cardiotoxicity or damage to other organs like the brain, liver and kidneys. Its administration can be improved by controlled delivery from agents like our hydrogels. Its structure consists of a hydrophobic anthraquinone ring connected to a more hydrophilic daunosamine group [26]. It is very poorly water-soluble, and solubility is increased as a hydrochloride salt. This amphiphilic nature requires a thoughtful design of the potential carrier to maximize DOX loading and release.

The different stability and hydrophilic–hydrophobic balance of each family of thermogels required a different approach to maximize DOX loading. In brief, CBS thermogels were loaded through diffusion after crosslinking, while dPG hydrogels were loaded in situ during network formation since drug incorporation after gelation led to network degradation. The different loading approach will probably affect loading efficiency and release kinetics, and it is an additional parameter to consider in the following discussion. For the more hydrophobic CBS family, the hydrogel was immersed in a saturated solution of DOX in ethanol (5 mg/mL) for 30 min at r.t. Then, the gel was removed from the vial, and the solvent was evaporated. For the hydrophilic dPG hydrogels, DOX was loaded during the formation of the network to enhance the affinity and increase the amount of loaded drug. The reagents and DOX were mixed in a THF/H_2_O solution and then exposed to UV light for crosslinking. DOX loading was quantified through HPLC (Table 1). Hydrogels **H2**, **H3** and **H5** showed similar loading capacity (0.4–0.5 mg per 100 mg of hydrogel) while **H4** doubled the loaded amount (0.8 mg/100 mg of hydrogel). This could be ascribed to a higher interaction with the dendritic network, for example, through H-bond interactions with the dPG crosslinkers and the acrylate groups.

**Figure 7:**
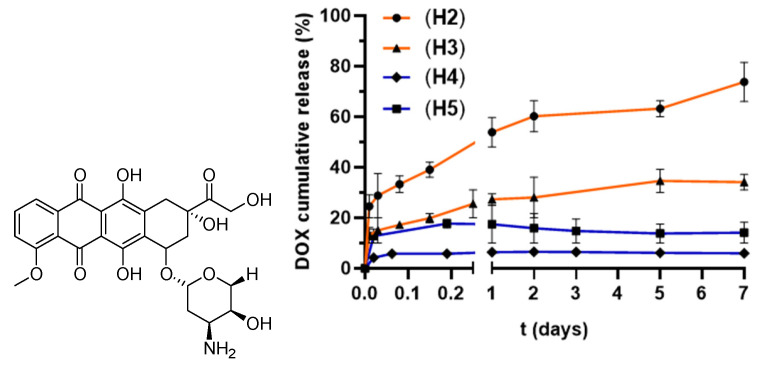
DOX release over time for CBS hydrogels **H2** and **H3**, and dPG hydrogels **H4** and **H5** at 37 °C, in percentage compared to the amount of loaded DOX in each hydrogel. Results are reported as mean ± standard deviation, in duplicate for two independent experiments.

DOX release in water solution (pH 7.4) was studied at 37 °C for one week. As shown in Figure 7 and Figure S5, a different behavior was observed for the two families. Polyglycerol thermogels showed a burst release of DOX during the first hours, reaching a maximum in the first 4–24 h and then reaching a plateau. In this case, DOX release is led by network cleavage. **H4**, and especially **H5**, are rapidly fractured in water solution, and thus most release is accomplished in the first hours, arising from the more exposed DOX. DOX trapped in the pores presents a high interaction with the network, and its release is not favored, keeping it trapped in the gel. These hydrogels showed less than 20% of drug release from the network. On the contrary, carbosilane hydrogels **H2** and **H3** showed a sustained release of DOX for 7 days, led by swelling, as DOX release closely resembles SD% curves. At the end of the experiment, carbosilane thermogels duplicated the amount of maximum released DOX, in the range 80–96 mg/L (Figure S5). The release is faster for **H2** than **H3**, reaching 75% and 35% release, respectively, after one week. Nevertheless, these results should be considered from a qualitative point of view, as the DOX loading strategy is not directly comparable between the two hydrogel families and may affect the release kinetics.

Preliminary kinetic analysis of DOX release was performed to understand the differences between the thermogels. Data were fitted to different drug release models such as first-order, Higuchi and Korsmeyer–Peppas models (Figure S6) [27,28]. The Higuchi model describes diffusion-controlled release from solid matrices, while the Korsmeyer–Peppas equation is commonly applied to polymeric systems to determine the transport mechanism through the release exponent “n”. Carbosilane hydrogels were closely fitted by the Korsmeyer–Peppas model, with R^2^ 0.96 (**H2**) and 0.97 (**H3**). In this model, the slope value “n” provides information about the release mechanism. For disk-shaped formulations, n = 0.5 indicates dominant Fick diffusion. In our case, we obtained n = 0.23, which can be interpreted as the drug release from the hydrogels is mainly governed by Fickian diffusion but with a restricted DOX mobility due to dense or rigid networks, resulting in a diffusion-controlled release mechanism. These data also fitted Higuchi model with two different slopes: a first burst release step with R^2^ 0.996 (**H2**, kH1 = 0.52) and 0.959 (**H3,** kH1 = 0.31), related to initial swelling, and a second controlled release step from the matrix, with R^2^ 0.95 (**H2**, kH2 = 0.64) and 0.88 (**H3**, kH2 = 0.26). Breakpoint analysis identified a transition at t = 6 h, separating the initial rapid diffusion phase from the subsequent diffusion-controlled release through the hydrogel network. For dPG hydrogels, none of the tested models fitted the data adequately, probably because they mainly underwent a fast burst release followed by the trapping of the drug within the gels.

## 4. Discussion

Dendritic hydrogels are a growing field that is poised for impactful translation advances in biomedicine. These networks exhibit unique and tunable physicochemical properties, especially promising for drug delivery, tissue engineering and wound-healing applications. During the last 25 years, several works have demonstrated the direct impact of dendritic architecture on hydrogel performance. The presence of dendritic crosslinkers, with multivalent branched architecture, provides high mechanical strength, customizable mesh size and sophisticated control over drug release. Additionally, they provide precise and batch-to-batch control over network formation. A step forward in the field of dendritic hydrogels is the design of stimuli-responsive networks, like the thermogels herein described.

As we have previously reported [9], three main components tailor the design of the hydrogels as well as their properties: the linear polymer, the dendritic crosslinker and the crosslinking reaction. For comparative purposes, in this work we employed the same polymer PLUL35(SH)_2_ as well as the same TEC reaction, while in the latter the conditions had to be adapted for the nature of the crosslinker. Carbosilane dendrimers are smaller entities with a high hydrophobic nature; on the contrary, dPGs are bigger structures with a more hydrophilic nature, only presenting 5% of alkene groups to react. This hampers the accomplishment of full crosslinking when reacting with PLUL35(SH)_2_ and may be responsible for the lower stability of dPG networks.

Our studies confirmed that we can tailor the properties of the networks. For example, the carbosilane dendrimers SiG0V_4_ (**1**), SiG1V_8_ (**2**) and SiG2V_16_ (**3**) showed that the increase in dendrimer generation led to a greater mechanical modulus, increased rigidity, finer mesh size and lower swelling degrees in the resultant hydrogels **H1**–**H3**. Additionally, they showed the impact of the hydrophilic–hydrophobic balance (HLB) between the dendritic crosslinker and the linear polymer to accomplish efficient crosslinking: longer reaction times are required to accomplish comparable GF for the more hydrophobic G1 and G2 dendrimers. We could fine-tune the swelling of CBS hydrogels by either altering the dendritic generation or the temperature. Around 50% shrinking of the hydrogel was observed when switching from the G2 to G3 crosslinker, and also when increasing the temperature from 25 °C to 37 °C. The presence of short-chain Pluronic fragments in the network supports the nanostructuring of the hydrogel and the modulation of swelling properties. Furthermore, the drug release, herein exemplified with doxorubicin, closely resembles the swelling pattern, thus connecting both events. Carbosilane hydrogels **H2** and **H3** showed a sustained release of DOX for 7 days, led by the swelling of the network. This release could be further modulated by the cleavage of the hydrogels in the presence of esterases, as the degradation study confirmed.

The design of dPG–Pluronic thermogels **H4** and **H5** under similar conditions was more challenging. They also exhibited a promising DOX loading and release, especially the acrylate hydrogel **H4**, which duplicated the amount of DOX loaded in the network. In this case, the DOX release pattern correlates to network fragmentation and reaches a maximum in the first 4–24 h. These hydrogels exhibited a fast cleavage in PBS at 25 °C, potentially explained by an incomplete gelation due to side reactions in the crosslinking step.

According to the mechanical properties of our hydrogels, with G′ >> G″ over the entire frequency range, they behave as soft viscoelastic solids. This agrees with other photo-crosslinked Pluronic hydrogels, with G′ values in the range of 1000–10,000 Pa [29,30]. In this case, carbosilane hydrogels are much stiffer networks with values above 80,000 Pa, and even higher for polyglycerol hydrogels, which are over 300,000 Pa. The dendritic generation and the temperature also modulate the mechanical properties. The temperature rise from 25 °C to 37 °C produces a decrease in the elastic modulus G′, probably due to the increase in hydrophobic interactions inside the Pluronic polymers.

The dendritic thermogels described herein appear as attractive alternatives to other temperature-responsive networks. The use of LCST polymers, such as Pluronics and PNIPAM, which become immiscible upon heating, produces hydrogels that swell at lower temperatures. As we demonstrated, the swelling degree can be fine-tuned by wisely selecting the network components. For example, Navarro et al. generated dPG-PNIPAM nanogels whose flexibility and size were modulated by parameters such as the size of crosslinker (5 vs. 10 kDa dPG) or the dPG:NIPAM ratio [31]. High amounts of dPG produced denser and more rigid networks with little volume change over the transition temperature, thus decreasing the responsiveness to temperature. On another example, Shymborska et al. attached a multiarm-PEG-based hydrogel nanocoating to temperature-responsive p(OEGMA-co-HEMA) brushes [32]. This enabled the development of smart polymer sandwiches, which underwent a wettability transition driven by the LCST of OEGMA units at around 20 °C. At T > T_c_, the brushes undergo a coil–globule transition, modulating the size of the pores within the nanocoating and expelling the water outside the hydrogel. Nevertheless, to maximize structural control in the hydrogel, we used a preformed LCST polymer like Pluronic L35. Pluronic polymers are a versatile family, with a myriad of commercially available polymers with different lengths and HLB. Our previous studies showed the crucial impact of the linear polymer to accomplish high-swelling hydrogels with temperature-responsiveness [9]. Pluronic L35 appeared as a promising candidate to generate carbosilane dendritic hydrogels, which showed SD 190% at 25 °C, while the counterparts L31 and L61 were too hydrophobic to enable swelling in water. However, PLU L35 and L61 were used to generate carbosilane dendritic–linear–dendritic hybrids that self-assembled into dendritic nanogels [33]. In this case, both polymers led to DNGs with promising antitumor activity, but higher in the case of the more hydrophobic L61.

Temperature responsiveness can also be accomplished using dendronized polymers. Ding et al. designed dendronized gelatins with pendant dendritic oligoethylene glycol (OEG) units, which exhibited LCST-type and UCST-type thermoresponsive behavior, depending on the grafting ratio of the dendritic OEGs [34]. The mechanical strength of the networks was modulated by either polymer concentration or grafting ratios of OEG dendrons. Similarly, dendronized copolymers containing OEG dendrons and terpyridine moieties showed both thermally induced reversible shrinking-swelling and redox-mediated gel–sol transitions by the addition of metal ions like Fe^2+^, highlighting the role of metallo-supramolecular chemistry in the design of advanced thermogels [35]. However, the presence of dendrimers or hyperbranched polymers like the ones herein employed improves the control over the network preparation and over the final properties of the thermogel, which is highly desired for biomedical applications.

It is well known that the phenomenon of critical solution temperature (CST)-based transitions arises from the formation of dynamic hydrogen bonds and hydrophobic interactions that behave differently at various temperatures [36]. In Pluronic polymers, the sol-to-gel transition is driven by the micellization of the PEO-PPO-PEO copolymer into closely packed cubic structures at the LCST. Initially, as the temperature rises, hydrophobic PPO blocks dehydrate and form spherical micelles with a PPO core and a PEO shell. With further heating, these micelles pack into ordered liquid crystalline structures, trapping water and forming a gel. In the dendritic thermogels herein described, the gelification of Pluronic chains is restricted due to the covalent attachment to the dendritic crosslinkers. However, the presence of the crosslinkers influences the temperature response. CBS dendrimers are highly hydrophobic crosslinkers that can promote the formation of hydrophobic pockets upon an increase in temperature (Figure S4). Indeed, they showed an increased rigidity (higher G′) with increasing generation, probably due to the increased hydrophobic interactions between the PPO fragments of Pluronic and the dendrimer. On the other hand, dPG crosslinkers present a highly hydrophilic scaffold with multiple hydroxyl groups that can establish dynamic hydrogen bonds with water, thus promoting hydration, or even with Pluronic terminal groups and forming loops that disfavor thiol-ene crosslinking. Our studies confirmed that dPG thermogels exhibited higher G′ values than carbosilane hydrogels, and the acrylate-containing hydrogel **H4** is stiffer than the allyl-functional hydrogel **H5**. This points to the potential H-bond formation in dPG networks.

## 5. Conclusions

Dendritic hydrogels represent a versatile platform for biomedical applications, offering tunable mechanical properties, swelling behavior, and drug release profiles. Our study demonstrates that the nature of the dendritic crosslinker strongly influences network performance. Carbosilane dendrimers allowed precise modulation of the mechanical properties of the hydrogels, mesh size, and swelling, leading to sustained doxorubicin release over several days, which could be further adjusted via enzymatic degradation. In contrast, dPG Pluronic thermogels presented higher hydrophilicity and lower stability, resulting in faster cleavage and burst release, highlighting the importance of crosslinker chemistry in network formation and stability.

Temperature responsiveness was effectively achieved using LCST-polymer Pluronic L35, with the swelling and mechanical properties finely tuned by the interplay between dendritic crosslinker, linear polymer, and reaction conditions. Overall, these findings underscore the potential of dendritic hydrogels—particularly carbosilane and polyglycerol architectures—as customizable carriers for drug delivery, where crosslinker selection, dendrimer generation, and polymer composition can be strategically designed to control hydrogel performance and responsiveness for biomedical applications. Future research directions include exploring hybrid dendritic architectures combining hydrophobic and hydrophilic crosslinkers, exploring the loading and release profile of other types of cargo such as macromolecules, and investigating in vitro and in vivo performance for controlled drug delivery, enabling tailored biomedical applications with improved efficacy and safety.

## Figures and Tables

**Table 1:** Relevant parameters for the dendritic thermogels **H1**–**H5**.

Crosslinker	M_w_ (g/mol)	Thermogel	GF (%)	Max. SD (%) 25 °C/37°C	G′(Pa) ^c^	Mesh Size (nm)	Loaded DOX (mg/100 mg gel)
SiG0V_4_ (**1**)	136.3	**H1**	95 ^a^	^b^	98,600	4.30	^c^
SiG1V_8_ (**2**)	585.3	**H2**	57 ^a^	400/220	81,300	2.13	0.420
SiG2V_16_ (**3**)	1483.2	**H3**	62 ^a^	230/120	340,000	2.85	0.491
dPG_10k_(OH)(ac) (**4**)	~10,000	**H4**	^b^	^b^	517,000	2.47	0.798
dPG_10k_(OH)(al) (**5**)	~10,000	**H5**	^b^	^b^	451,500	2.59	0.442

^a^ After 1.5 h irradiation. ^b^ Not measurable. ^c^ Measured in a frequency sweep assay, at 1% frequency.

## Data Availability

The original contributions presented in this study are included in the article/Supplementary Material. Further inquiries can be directed to the corresponding author.

## Supplementary Materials

The following supporting information can be downloaded at: https://www.mdpi.com/article/10.3390/pharmaceutics18030362/s1. Figure S1. ^1^H-NMR of dPG-acrylate **4**. Figure S2. ^1^H-NMR of dPG-allyl **5**. Figure S3. Comparative DLS study of PLUL35 and PLUL35(SH)_2_ at 25 °C. Figure S4. Proposed mechanisms for the thermoresponsive behavior of thermogels. Figure S5. DOX release over time for hydrogels **H2–H5** at 37 °C. Figure S6. Fitting of DOX release data from hydrogels **H2–H5** to different kinetic models.
