# Supplementary material for: Tailoring the Design of Dendritic Thermogels Through Carbosilane and Polyglycerol Crosslinkers
Source: Pharmaceutics. 2026 Mar 13;18(3):362. doi: 10.3390/pharmaceutics18030362 (PMC13029497; doi:10.3390/pharmaceutics18030362)
Supplement: Supplementary file 1 [file pharmaceutics-18-00362-s001.zip › pharmaceutics-4160673-supplementary.pdf]

Electronic Supporting Information

Tailoring the Design of Dendritic Thermogels Through Carbosilane and Polyglycerol Crosslinkers

Judith Recio-Ruiz [1], Boonya Thongrom [2], F. Javier de la Mata [1,3,4], Rainer Haag [2] and Sandra García-Gallego [1,3,4,*]

[1] University of Alcala, Department of Organic and Inorganic Chemistry and Research Institute in Chemistry "Andrés M. Del Río" (IQAR), 28805 Madrid, Spain
[2] Institute for Chemistry and Biochemistry, Freie Universität Berlin, Takustr. 3, 14195 Berlin, Germany
[3] Networking Research Center on Bioengineering, Biomaterials and Nanomedicine (CIBER-BBN), 28029 Madrid, Spain
[4] Institute Ramón y Cajal for Health Research (IRYCIS), 28034 Madrid, Spain

TABLE OF CONTENTS

Figure S1. ^{1}H-NMR of dPG-acrylate **4** …….……………………………………………………………2

Figure S2. ^{1}H-NMR of dPG-allyl **5** …….……………………………………………………………………3

Figure S3. Comparative DLS study of PLUL35 and PLUL35(SH)$_2$ at 25 °C………………………3

Figure S4. Proposed mechanisms for the thermoresponsive behavior of thermogels...4

Figure S5. DOX release over time for hydrogels **H2-H5 a**t 37°C……………………………………5

Figure S6. Fitting of DOX release data from hydrogels **H2-H5** to different kinetic models..5

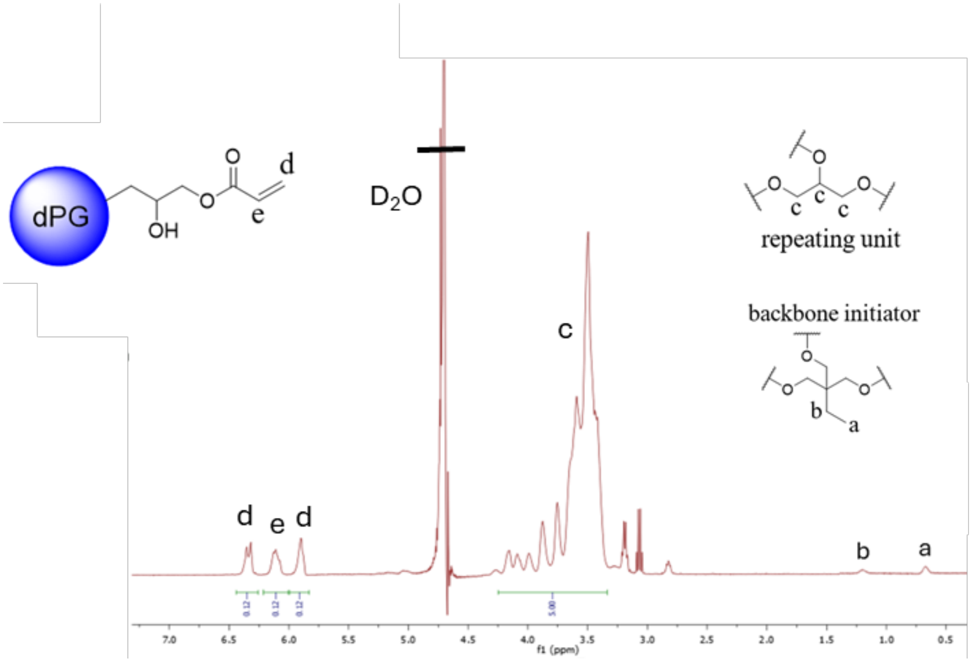

Figure S1. ^{1}H-NMR (500 MHz, D_2O, δ (ppm)) of dPG-acrylate **4** (5%).

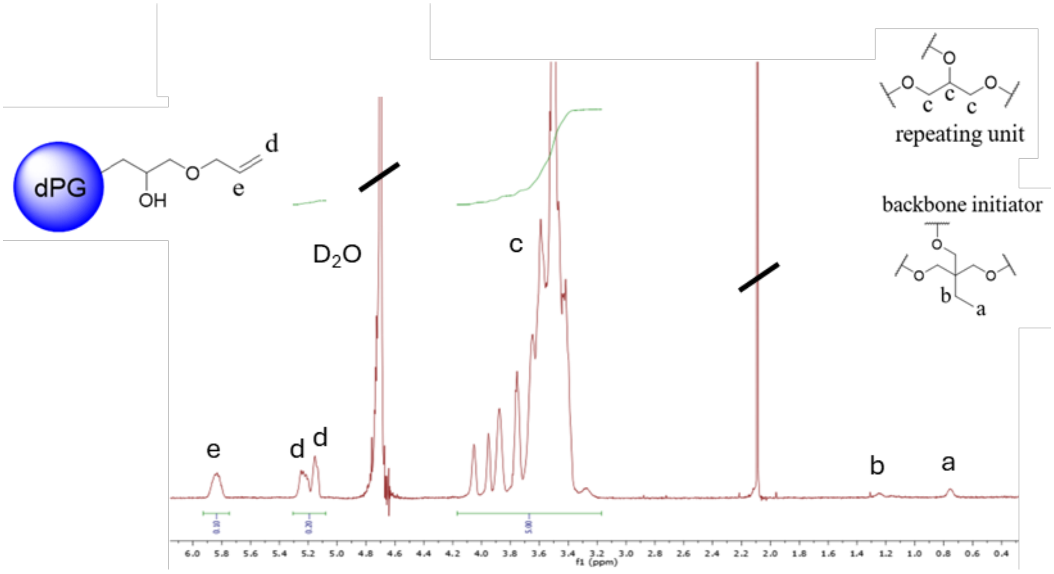

Figure S2. ^{1}H-NMR (500 MHz, D_2O, δ (ppm)) of dPG-allyl **5** (5%).

PluL35 6 mM

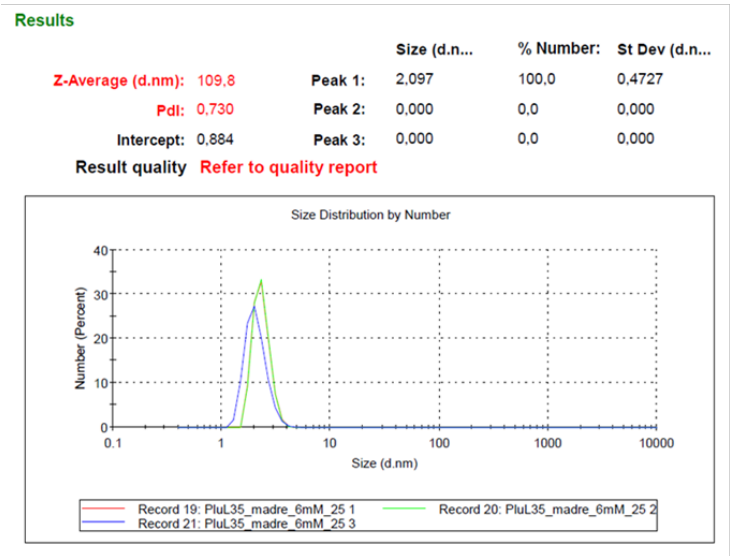

PluL35(SH)$_2$ 6 mM

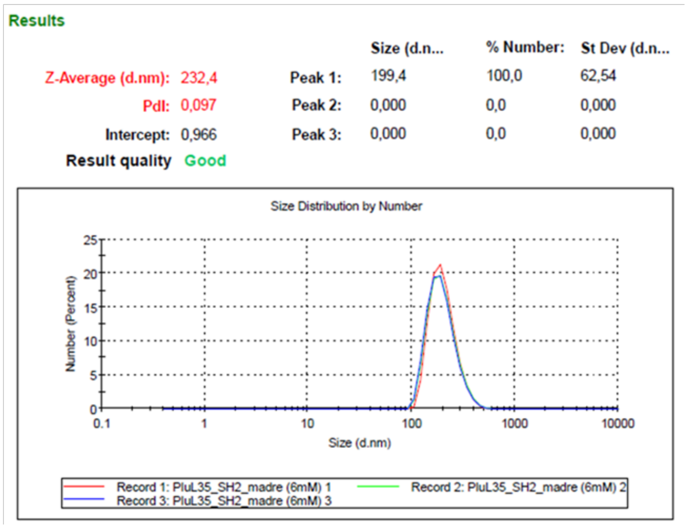

Figure S3. Comparative DLS study of PLUL35 and PLUL35(SH)$_2$ at 25 °C.

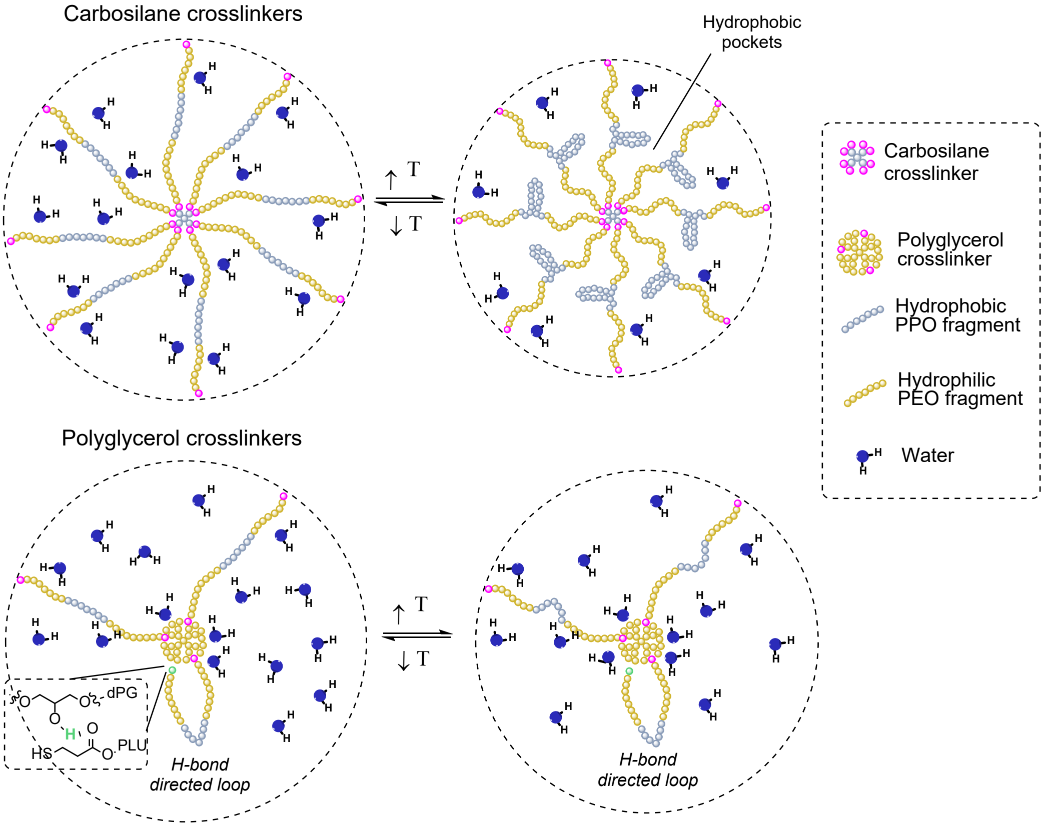

Figure S4. Proposed mechanisms for the thermoresponsive behavior of dendritic thermogels. Top: In CBS thermogels, the increase in temperature produces the dehydration of PPO fragments, which tend to form hydrophobic pockets together with the hydrophobic carbosilane crosslinkers. Bottom: In dPG thermogels, the presence of multiple hydroxyl groups in the dPG crosslinker promotes hydration through H-bond formation. Additionally, it can generate H-bond directed loops of Pluronic chains.

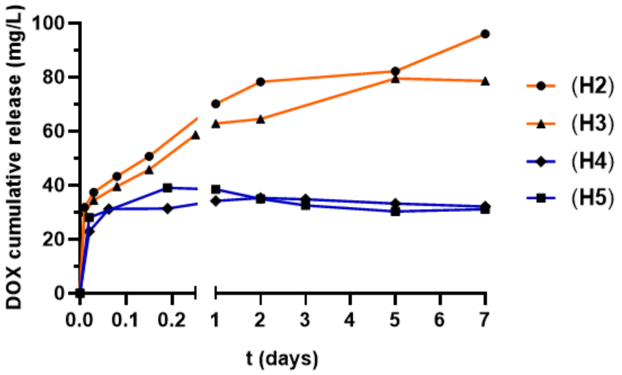

Figure S5. DOX release over time for CBS hydrogels **H2** and **H3**, and dPG hydrogels **H4** and **H5** at 37°C. Hydrogels weight and loaded DOX: **H2** (32.0/0.13 mg), **H3** (47.6/0.23 mg), **H4** (50.8/0.54 mg) and **H5** (68.1/0.22 mg). Average results from two independent experiments.

A) First-order fitting

$$\ln(100 - Q_t) = \ln 100 - k_1 t$$

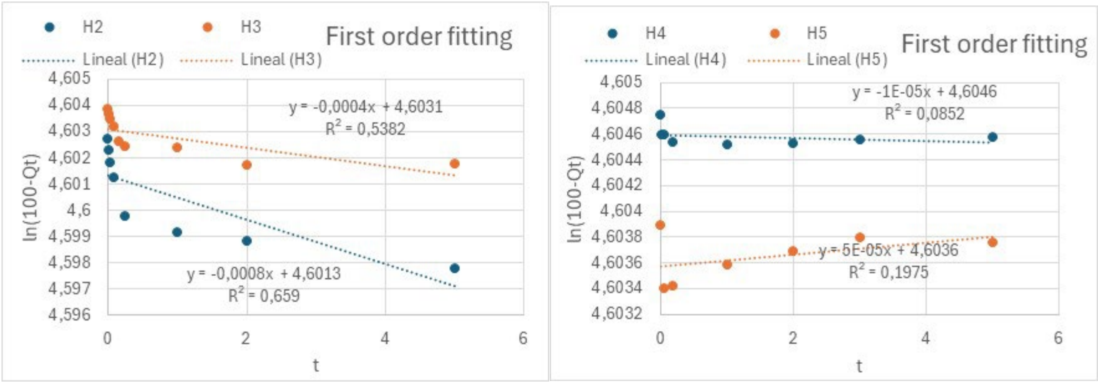

B) Higuchi fitting

$$Q_t = k_H \sqrt{t}$$

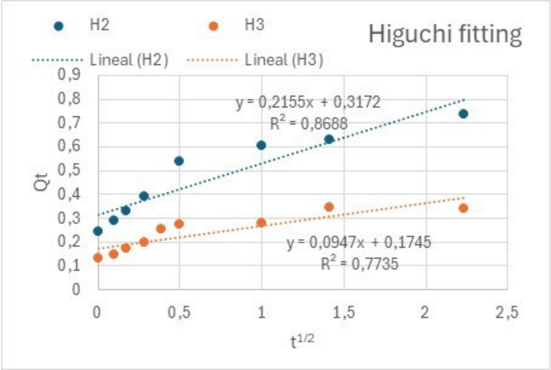

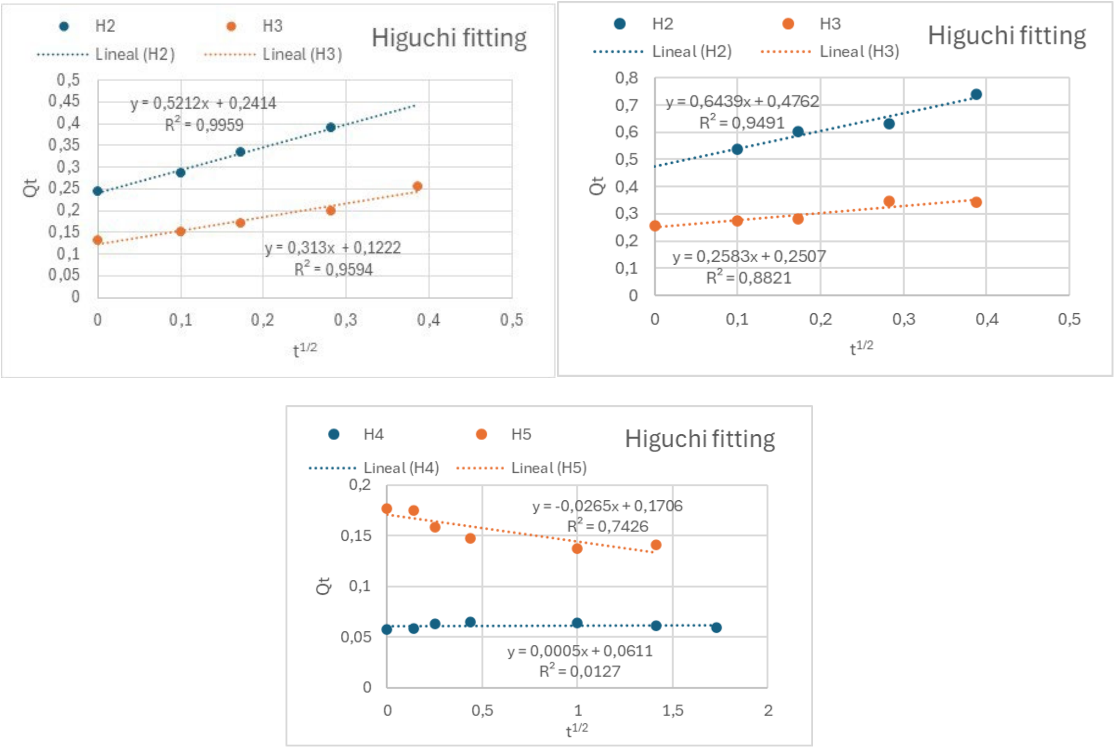

C) Korsmeyer-Peppas fitting

$$\log(M_t/M_\infty) = \log k + n \log t$$

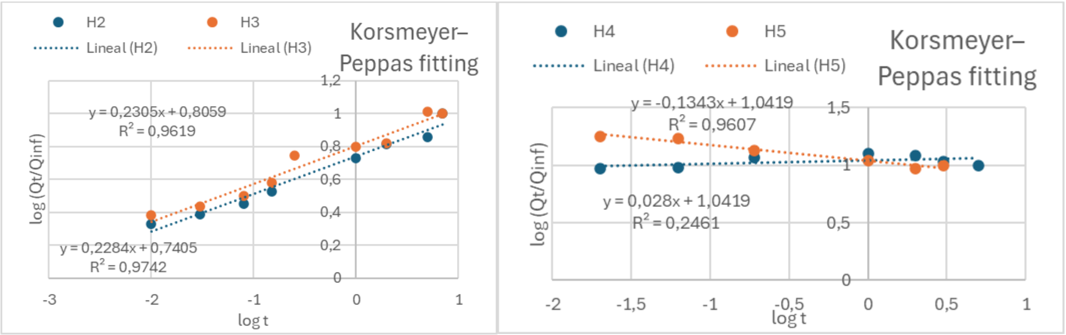

Figure S6. Fitting of DOX release over time for CBS hydrogels **H2** and **H3**, and dPG hydrogels **H4** and **H5**. In all models, t=0 was removed from fitting. a) First-order model. b) Higuchi model. For H2-H3, model was fitted to two different lines (burst release R^2 0.996, and controlled release R^2 0.959). For H4-H5, initial burst release points were removed from fitting. c) Korsmeyer-Peppas model. For H2-H3, model was fitted to a single line (R^2 0.961 and 0.974). For H4-H5, initial burst release points were removed from fitting.
